# Effects of orally administered galacto-oligosaccharides on immunological parameters in foals: a pilot study

**DOI:** 10.1186/s12917-014-0278-4

**Published:** 2014-11-19

**Authors:** Johannes Cornelis Vendrig, Luc Edgar Coffeng, Johanna Fink-Gremmels

**Affiliations:** Veterinary Pharmacology, Pharmacotherapy and Toxicology, Institute for Risk Assessment Sciences, Faculty of Veterinary Medicine, Utrecht University, P.O. box 80152, 3508 TD Utrecht, The Netherlands; Department of Public Health, Erasmus MC University Medical Center Rotterdam, P.O. box 3000, 2040 CA Rotterdam, The Netherlands

**Keywords:** Horse, Foal, Oligosaccharides, PBMC, GOS, Immunomodulation, Cytokines, Bayesian Hierarchical linear regression

## Abstract

**Background:**

In the first phase of life, in which the immune system is primed and the bacterial colonization of epithelial surfaces takes place, foals are highly susceptible to bacterial infections. Next to strategies to optimize maternally acquired immunity in individual foals, current research explores other options to modulate immune responses in foals. During the past decades, oligosaccharide supplements were developed to mimic beneficial properties of the oligosaccharides, which are present in colostrum and milk. In human infants and laboratory animal species, dietary supplementation with galacto-oligosaccharides (GOS) has been shown to result in prebiotic and immunomodulating effects, with long-term beneficial consequences for both defensive and allergic immune responses. As yet, no studies are published concerning the *in vivo* effects of GOS in horses. The current study was designed as a pilot study to investigate the effects of an orally applied, commercially available GOS product in a group of pony foals. The treatment and the control group consisted of six and four foals, respectively. Foals were treated during the first four weeks of life and subsequently followed up for another ten weeks.

**Results:**

In peripheral blood mononuclear cells (PBMCs) derived from GOS-treated foals at day 28, a standardized lipopolysaccharide challenge resulted in significantly lower relative mRNA expression levels of the pro-inflammatory cytokines interferon-γ and interleukin-6 compared with PBMCs of control foals. In the 98-day period of investigation, no significant effects of the GOS supplement were observed on clinical and blood parameters for immunity and general health in these foals.

**Conclusions:**

Based on these first results, we can conclude that this dose regimen of GOS was well accepted by the foals and did not result in any detectable undesirable side effects. More clinical trials are required to confirm the attenuating effects of GOS treatment on equine pro-inflammatory immune responses and to implement this into practice.

**Electronic supplementary material:**

The online version of this article (doi:10.1186/s12917-014-0278-4) contains supplementary material, which is available to authorized users.

## Background

With birth, the foal’s immune system is subjected to a range of environmental challenges. Bacterial colonization of epithelial surfaces is one of the major events in early life determining immune responses in later phases of life [1]. In addition to the basic concept of protection by maternal antibodies present in colostrum, recently more interest has arisen for the influence of other colostral constituents, such as oligosaccharides. In human infants and experimental animals, dietary supplements containing commercially produced galacto-oligosaccharides (GOS) have been proven to elicit prebiotic effects [2–4] and to be beneficial for health in several ways [5], for instance by enhancing defensive immune responses [6–8] and lowering the incidence of infections [9,10]. Still, the evidence supporting the use of such oligosaccharide supplements is not conclusive, as other studies have reported only temporary or non-significant effects of similar supplements on infection and immunity [11–13].

A limited amount of research into the effects of oligosaccharides in horses has been conducted so far. Prebiotic properties of short-chain fructo-oligosaccharides in horses have been confirmed [14,15], but to our knowledge no studies into *in vivo* effects of GOS or other oligosaccharide supplements in horses or foals have been published. Our previous *in vitro* studies with equine peripheral blood mononuclear cells (PBMCs) revealed immunomodulatory properties of both equine colostral carbohydrates and several commercially available oligosaccharide products, including GOS [16,17].

The present study was designed as a pilot study to investigate the *in vivo* effects of a commercially available GOS product in young foals. Next to assessing the safety of the chosen dose regimen of GOS and its effects on immunity and general health, we compared the responses to a standardized lipopolysaccharide (LPS) challenge in *ex vivo* PBMCs after 4 weeks of treatment.

## Methods

### Foals

Twelve warmblood pony foals (crossbreed New Forest*Arabian) were included in the study, all born at a horse dairy farm in The Netherlands, where the experiment was carried out. All mares and foals were housed together as a group under identical and stable conditions. Foals were born by natural delivery within a period of ten weeks and were allocated to the treatment group and the control group at random. In total, six foals were supplemented with GOS and four foals were included in the control group (two foals were excluded during the course of the study: see results section). Foals were naturally nursed by the mares and were also free to eat roughage (hay, grass, straw) as soon as they were ready for it. All experimental procedures were approved by the committee of ethical considerations in animal experiments of Utrecht University (DEC Utrecht, Permit Number: 2012.III.05.053).

### Dietary supplementation

Foals in the treatment group were orally supplemented with GOS for 28 days, starting at the day of birth. The applied GOS product (Vivinal GOS syrup, FrieslandCampina Domo, Zwolle, The Netherlands) consisted of approximately 45% GOS, 16% lactose, 14% glucose, and 25% water. The product contained short chain GOS, which are oligomers (degree of polymerization 2–6) constructed from lactose [18]. An amount of 15 g GOS syrup was supplemented twice daily. Foals in the control group were orally supplemented with 2.4 g lactose and 2.1 g glucose (twice daily for 28 days), resembling the amounts of glucose and lactose that the GOS treated foals received through the GOS syrup. Both the GOS syrup and the control treatments were administered directly into the mouth of the foals (with a syringe).

### Sampling and output parameters

At day 0, 1, 7, 14, 21, 28, 42, 56, 70, 84, and 98, approximately 14 mL of blood was collected by jugular venipuncture directly into sterile blood collection tubes; one 10 mL serum tube and one 4 mL spray-coated EDTA tube (BD Vacutainer Systems, Plymouth, United Kingdom). At all time points, samples were sent to the University Veterinary Diagnostic Laboratory in Utrecht to determine red and white blood cell parameters (including hematocrit, leukocyte count, and leukocyte differentiation) and the total protein concentrations including a protein electrophoresis (albumin and differentiation of globulins). In addition, aliquots of the serum samples were stored at −80°C to perform several ELISAs. Serum concentrations of the immunoglobulin subtypes IgG(a), IgG(b), IgG(T), IgM and IgA were quantified, using commercial ELISA kits according to the manufacturer’s instructions (Bethyl Laboratories Inc., Montgomery, AL, USA). Mare colostrum was sampled within 12 hours postpartum to investigate the concentrations of immunoglobulin subtypes by means of the same ELISA kits (Bethyl Laboratories Inc., Montgomery, AL, USA).

### PBMC experiments

At day 28, the last day of GOS application, PBMCs were isolated from each foal according to previously described methods [16]. PBMCs were resuspended in RPMI 1640 Medium (Lonza, Basel, Switzerland) containing 2 mM glutamine (Lonza, Basel, Switzerland), 100 IU/mL penicillin (Lonza, Basel, Switzerland), 100 μg/mL streptomycin (Lonza, Basel, Switzerland) and 10% horse serum. Horse serum was prepared by collecting full blood, allowing it to clot for 45 minutes, and then collecting the serum following centrifugation. The serum for all experiments was prepared at once by collection of blood from one pony (not enrolled in the study) from the same herd.

PBMCs were seeded in 24 well plates at a density of 4*10^6^ cells/mL medium/well. Subsequently, the plates were incubated for 2 hours at 37°C and 5% CO_2_. The plates were then centrifuged for 10 minutes at 400× *g* before refreshing the medium without removing PBMCs. The experiments were started by replacing the medium with medium containing 0 or 1 μg/mL LPS (*Escherichia coli* O111:B4, Sigma-Aldrich, St. Louis, MO, USA). After 4 hours of incubation, supernatants were collected for ELISA and stored at −80°C; for qPCR, PBMCs were lysed using RNA lysis buffer (Promega, Madison, WI, USA) and stored at −80°C. All individual incubations were performed in triplicate for each foal.

Protein levels of tumor necrosis factor-α (TNF-α), interferon-γ (IFN**-γ**) and interleukin-10 (IL-10) were measured by means of ELISA on the cell culture supernatants, using Duoset® ELISA Development System for equine TNF-α, equine IFN**-γ,** and equine IL-10 (R&D Systems, Minneapolis, MN, USA). Standard operating procedures of the manufacturer were followed, applying all required buffers and solutions in the form provided by the manufacturer (R&D Systems, Minneapolis, MN, USA). The lower limits of detection of the ELISAs were 15.6 pg/mL, 62.5 pg/mL, and 156.3 pg/mL for TNF-α, IFN**-γ,** and IL-10, respectively.

RNA was isolated from PBMCs using SV Total RNA isolation system according to the manufacturer’s instructions (Promega, Madison, WI, USA). Isolated fractions were dissolved in 50 μL RNAse free water and stored at −80°C. Quality and quantity of RNA was determined spectrophotometrically (Nanodrop). cDNA was generated using iScript™ cDNA Synthesis Kit (Biorad, Hercules, CA, USA) according to the manufacturer’s protocol. For reverse transcriptase reaction, 1000 ng RNA was applied per sample. Expression of mRNA was assessed by real-time PCR using a Biorad iQ5 Multicolor Real-time PCR detection system and iQ™ SYBR® Green Supermix (Biorad, Hercules, CA, USA). Specific primer pairs were designed and tested for efficiency and accuracy, after having checked their specificity using the NCBI-BLASTN search program. Primer pairs were synthesized commercially (Eurogentec Nederland B.V., Maastricht, The Netherlands). For this study, mRNA expression of IFN**-γ**, IL-4, IL-6, IL-10, IL-13, IL-17, transforming growth factor-β1 (TGF-β1), TNF-α, β-actin, and glyceraldehyde 3-phosphate dehydrogenase (GAPDH) was determined using the following primer pairs:

- IFN-γ: F 5′-GATCTGAAGGTCCAGCGCAA-3′ R 5′-TCCGGCCTCGAAATGGATTC-3′;
- IL-4: F 5′-GCCCGAAGAACACAGATGGA-3′ R 5′-CAGTACAGCAGGTCCCGTTT-3′;
- IL-6: F 5′-TGGCTGAAGAACACAACAACT-3′ R 5′-GAATGCCCATGAACTACAACA-3′;
- IL-10: F 5′-GAGAACCACGGCCCAGACATCAAG-3′ R 5′-GACAGCGCCGCAGCCTCACT-3′;
- IL-13: F 5′-AGCAGTCATTGCTCTCGCTT-3′ R 5′-TGGGTGATGTTGACCAGCTC-3′;
- IL-17: F 5′-GCTGAGTCTGGTGGCTATCG-3′ R 5′-TTCTTGTCCCCAGTGTTCGG-3′;
- TGF-β1: F 5′-TGTCCACCTGCAAGACCATC-3′ R 5′-CCGCAACTTGGACAGGATCT-3′;
- TNF-α: F 5′-TCCAGACGGTGCTTGTGC-3′ R 5′-GGCCAGAGGGTTGATTGACT-3′;
- β-actin: F 5′-CAAGGCCAACCGCGAGAAGATGAC-3′ R 5′-GCCAGAGGCGTACAGGGACAGCA-3′;
- GAPDH: F 5′-TGGCATGGCCTTCCGTGTCC-3′ R 5′-GCCCTCCGATGCCTGCTTCAC-3′.

### Statistical analysis

Data were analyzed in a Bayesian framework. This means that we considered the data as fixed (which it is once observed) and parameters as random variables because they are unknown (in a frequentist framework, data are considered random and parameters are fixed). In other words, our inferences are conditioned on the observed data, and not on the distribution of test statistics across a range of unobserved repeated samples. Related to this, the Bayesian context facilitates a common-sense interpretation of uncertainty in parameter estimates [19]. For example, a posterior 95% credible interval of 1–10 means that the true value of the parameter lies between one and ten with 95% probability (conditional on the chosen model). Model selection can be done based on the deviance-based information criterion (and several other criteria), which can even be used to compare hierarchical models. Also, a Bayesian framework provides more robust parameter estimates, as such estimates are exact (we need not rely on asymptotic theory as we would in a frequentist framework) and we do not have to be concerned about multiple testing. These last two points are especially relevant to the current study (small sample size, many outcome parameters).

Data on blood parameters (day 28 separately, and the entire period from day 0–98) as well as PBMC data (day 28 only) were analyzed by means of Bayesian linear regression. Similar to previous analyses [17], PBMC responses were allowed to vary between horses in general (random intercept), and with regard to incubation with LPS (random slope). To stabilize the variance, all blood and PBMC data were log-transformed, except for hematocrit data, which were analyzed on the unit scale. For the analysis of the blood data over the entire period, time was entered into the model as a continuous variable. To account for the sometimes non-linear association between time and blood data, time was transformed by means of fractional polynomials, such that the statistical model best fitted the data. To minimize bias in blood data due to variable uptake of colostral globulins, the first weeks of life were not included in the analysis for β-2 and γ globulins (up to day 42), IgA and IgM (up to day 28), IgG(a) (up to day 42), and IgG(b) and IgG(T) (up to day 56). The selection of these periods was based on visual inspection of the data, and was made such that at worst, estimates of the difference between the control and GOS group would be conservative.

For each data type, the best fitting fractional polynomials were determined in R (version 2.15.3; [20]), using the package *mfp* (version 1.4.9; [21]), not yet accounting for variation between horses. Final models, including random intercepts and fractional polynomials, were analyzed in JAGS, a program for analysis of Bayesian models using Markov Chain Monte Carlo simulation (version 3.3.0; [22]). Simulations in JAGS were performed using R packages *rjags* (version 3–10; [23]) and *R2jags* (version 0.03-08; [24]). Posterior distributions were estimated based on uninformative prior distributions (normal distributions with mean 0 and standard deviation 100 for parameter means; inverse gamma distributions with mean 1 and variance 10,000 for variance of measurement error; uniform distributions with range 0 to 100 for standard deviation of random intercepts). Differences between control and GOS groups were stated significant, based on 95% Bayesian credible intervals, which were defined as the 2.5% and 97.5% percentiles of the posterior distributions. Posterior distributions were simulated by means of four Markov chains, each consisting of 10,000 Monte Carlo samples. The first 5,000 samples were discarded for burn-in, allowing the model to converge. Model convergence was assessed by means of Gelman and Rubin’s convergence diagnostic, the potential scale reduction factor [25].

### qPCR data validation

Variation of mRNA expression was excluded as a confounding factor, as no significant differences in either β-actin or GAPDH expression were detected between PBMCs from GOS-treated foals and foals from the control group, or between the blank incubations and the incubations with LPS.

In addition, we analyzed the data with a correction for GAPDH, according to the method described by Livak and Schmittgen [26]. In our case, normalization of data (based on housekeeping genes) did not change point estimates for differences between incubation types. Furthermore, normalization did not reduce the variation in the data (as should be expected in case of confounding due to variation in total mRNA levels), but rather increased it. This indicated that the error due to variation in mRNA expression, if any, was negligible compared to basic measurement error in the data. Based on this knowledge and the fact that housekeeping genes for equine PBMCs have not yet been validated, we decided to present the data in this manuscript without any corrections.

## Results

### Course of the study

During the 98-day period of investigation, no effects of the treatment on the apparent clinical health parameters were observed, either in the GOS-treated foals or in the control group. The treatments were well accepted by the foals and no undesirable side effects (including diarrhea) were encountered.

Two foals in the control group were excluded from the study; one foal was stillborn, and another foal was not treated on the first two days of life due to colic. Therefore, the results comprise the data of six GOS-treated foals and four foals from the control group.

### Blood parameters

**Figure 1:**
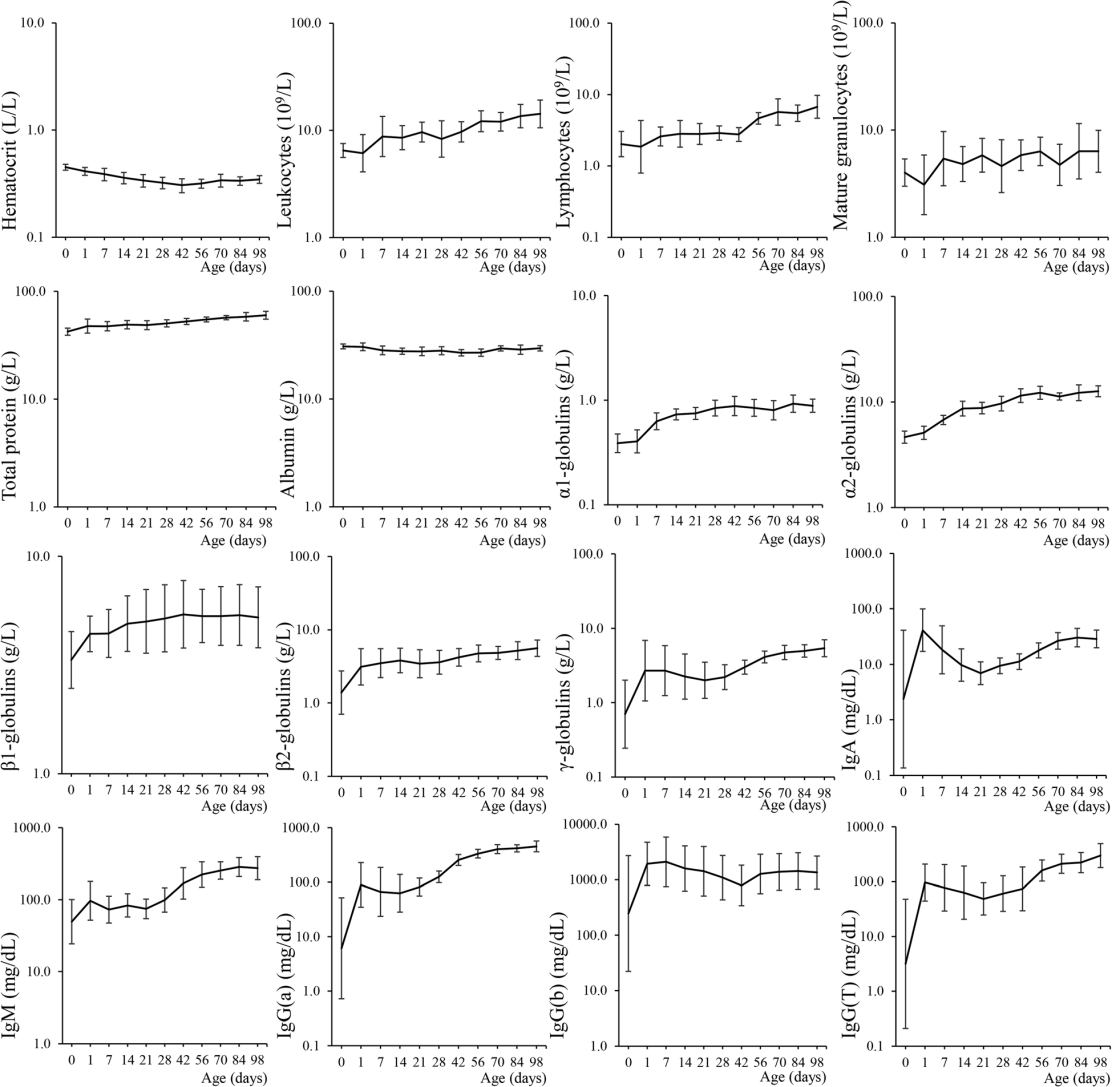
**Blood parameters.** Mean values and standard deviations for the investigated blood parameters at each time point for all foals included in the study.

Table 1 represents the comparison of the blood parameters between GOS-treated and control foals during the entire period of investigation. None of the blood parameters differed significantly between the two groups of foals. In Figure 1, a quantification of blood parameters is given for all foals at each time point for the entire period of investigation. Table 2 illustrates the comparison of the blood parameters between the two groups of foals at day 28 (at the moment of PBMC isolation). At day 28, the blood concentration of IgG(a) was found to be significantly higher in the group of GOS-treated foals compared to control group. Apart from this, no significant differences were observed between the two groups at day 28.

**Table 1:** **Blood parameters: comparison between GOS-treated and control foals throughout the 98 days of the experiment**

	**Difference between treatment and control group including 95% Bayesian credible intervals** ^**1**^		
	**Median**	**Lower bound**	**Upper bound**
Leukocytes	0.88	0.66	1.15
Lymphocytes	0.89	0.66	1.19
Mature granulocytes	0.89	0.62	1.28
Total protein^2^	1.03	0.94	1.13
Albumin (absolute values)	1.05	0.96	1.15
α-1 globulins (absolute values)	1.09	0.87	1.36
α-2 globulins (absolute values)	0.94	0.84	1.04
β-1 globulins (absolute values)	1.16	0.72	1.97
β-2 globulins (absolute values)	1.05	0.74	1.54
γ globulins (absolute values)	1.10	0.83	1.44
Albumin (% of total protein)	1.01	0.94	1.09
α-1 globulins (% of total protein)	1.05	0.83	1.36
α-2 globulins (% of total protein)	0.91	0.81	1.02
β-1 globulins (% of total protein)	1.09	0.74	1.61
β-2 globulins (% of total protein)	1.01	0.71	1.44
γ globulins (% of total protein)	1.07	0.87	1.30
IgA	1.02	0.66	1.55
IgM	1.20	0.72	1.99
IgG(a)	1.13	0.91	1.42
IgG(b)	1.77	0.66	4.99
IgG(T)	1.34	0.74	2.38

^1^Relative differences are stated (significant if ≠ 1). For the hematocrit, the absolute difference was 0.00 (interval ranging from −0.04 to 0.05). ^2^The protein spectrum (albumin and different globulins) are compared both as absolute values (g/L) and as a percentage of the total protein concentration.

**Table 2:** **Blood parameters: comparison between GOS-treated and control foals at day 28 (at the moment of PBMC isolation)**

	**Difference between treatment and control group including 95% Bayesian credible intervals** ^**1**^		
	**Median**	**Lower bound**	**Upper bound**
Leukocytes	0.85	0.47	1.57
Lymphocytes	0.83	0.60	1.15
Mature granulocytes	0.83	0.34	2.02
Total protein^2^	1.03	0.92	1.16
Albumin (absolute values)	0.99	0.87	1.14
α-1 globulins (absolute values)	1.01	0.77	1.33
α-2 globulins (absolute values)	1.06	0.82	1.36
β-1 globulins (absolute values)	1.18	0.68	2.02
β-2 globulins (absolute values)	1.14	0.63	2.01
γ globulins (absolute values)	1.11	0.61	2.02
Albumin (% of total protein)	0.96	0.87	1.08
α-1 globulins (% of total protein)	0.98	0.71	1.37
α-2 globulins (% of total protein)	1.03	0.81	1.29
β-1 globulins (% of total protein)	1.15	0.70	1.88
β-2 globulins (% of total protein)	1.10	0.62	1.95
γ globulins (% of total protein)	1.07	0.64	1.81
IgA	1.41	0.92	2.18
IgM	1.15	0.62	2.08
IgG(a) *	**1.43**	**1.10**	**1.83**
IgG(b)	2.14	0.58	7.99
IgG(T)	1.70	0.56	5.00

^1^Relative differences are stated (significant if ≠ 1, marked with asterisk). For the hematocrit, the absolute difference was −0.01 (interval ranging from −0.07 to 0.06). ^2^The protein spectrum (albumin and different globulins) are compared both as absolute values (g/L) and as a percentage of the total protein concentration.

An additional file is provided to illustrate the data in more detail for the individual foals in both groups (see [Additional file 1]).

### Colostral and serum immunoglobulins

The measured concentrations of immunoglobulin subtypes in the colostral samples of the individual mares are added to the immunoglobulin data of the corresponding foals in the provided additional file (see [Additional file 1]). No significant differences were observed with regard to concentrations of the measured immunoglobulin subtypes in the mares’ colostrum between the two groups. The colostral immunoglobulin levels for all mares exceeded the minimal quality standards for equine colostrum (for instance 3000 mg/dL for IgG [27]). Moreover, the colostrum of the dams of foals with relatively low serum globulin concentrations during the first weeks did not contain lower amounts of immunoglobulins relative to the colostrum of dams belonging to foals with high serum globulin concentrations.

The passive transfer of immunity at day 1 was within the expected range, as all foals reached serum IgG concentrations well above 800 mg/dL, except for one foal in the control group (i.e., 294 mg/dL 24 hours postpartum). In the analysis of endogenously produced immunoglobulins, no significant differences were found in the entire group of foals and the serum of the aforementioned foal reached comparable immunoglobulin concentrations as well (in the period >42 days of age, as can be observed in the graphs of [Additional file 1]).

### PBMC experiments

**Figure 2:**
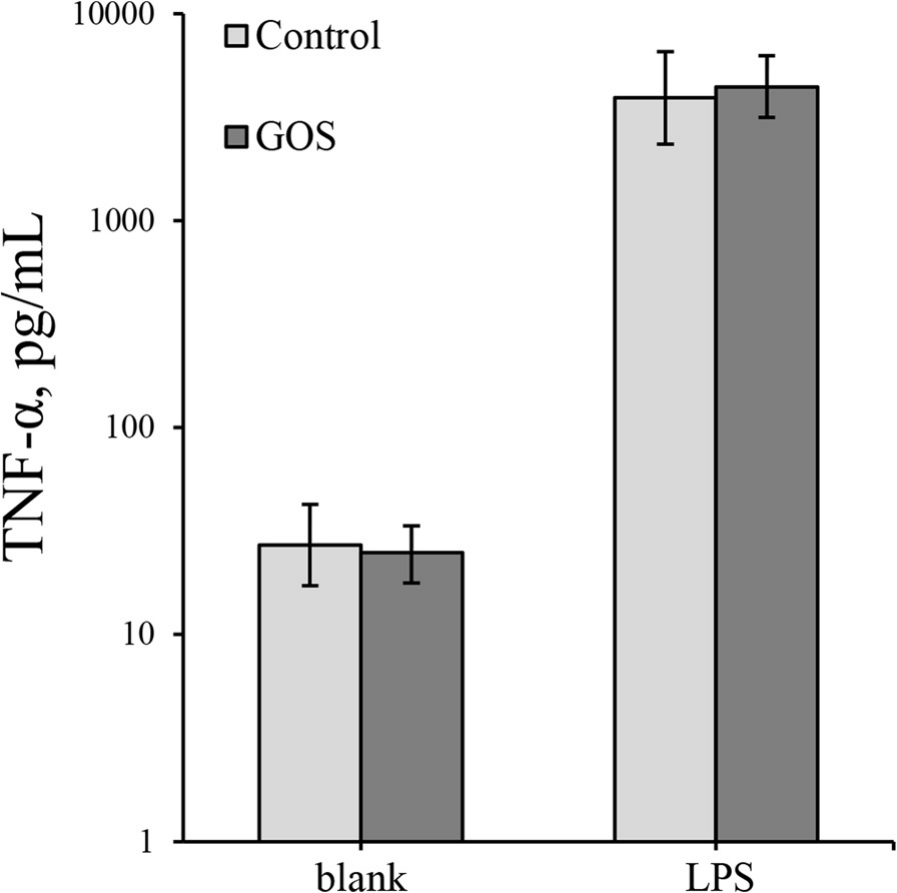
**TNF-α production.** Mean supernatant concentrations of TNF-α (in pg/mL) including 95% Bayesian credible intervals for unchallenged (blank) and LPS-challenged PBMCs derived from foals treated with either GOS or glucose/lactose (control).

Figure 2 illustrates supernatant concentrations of TNF-α for unchallenged and LPS-challenged PBMCs. No significant difference in TNF-α production was observed between the two groups. The data for supernatant concentrations of IL-10 and IFN-γ were mostly below the detection limit of the applied assay. Hence, analysis of these data was not possible.

**Figure 3:**
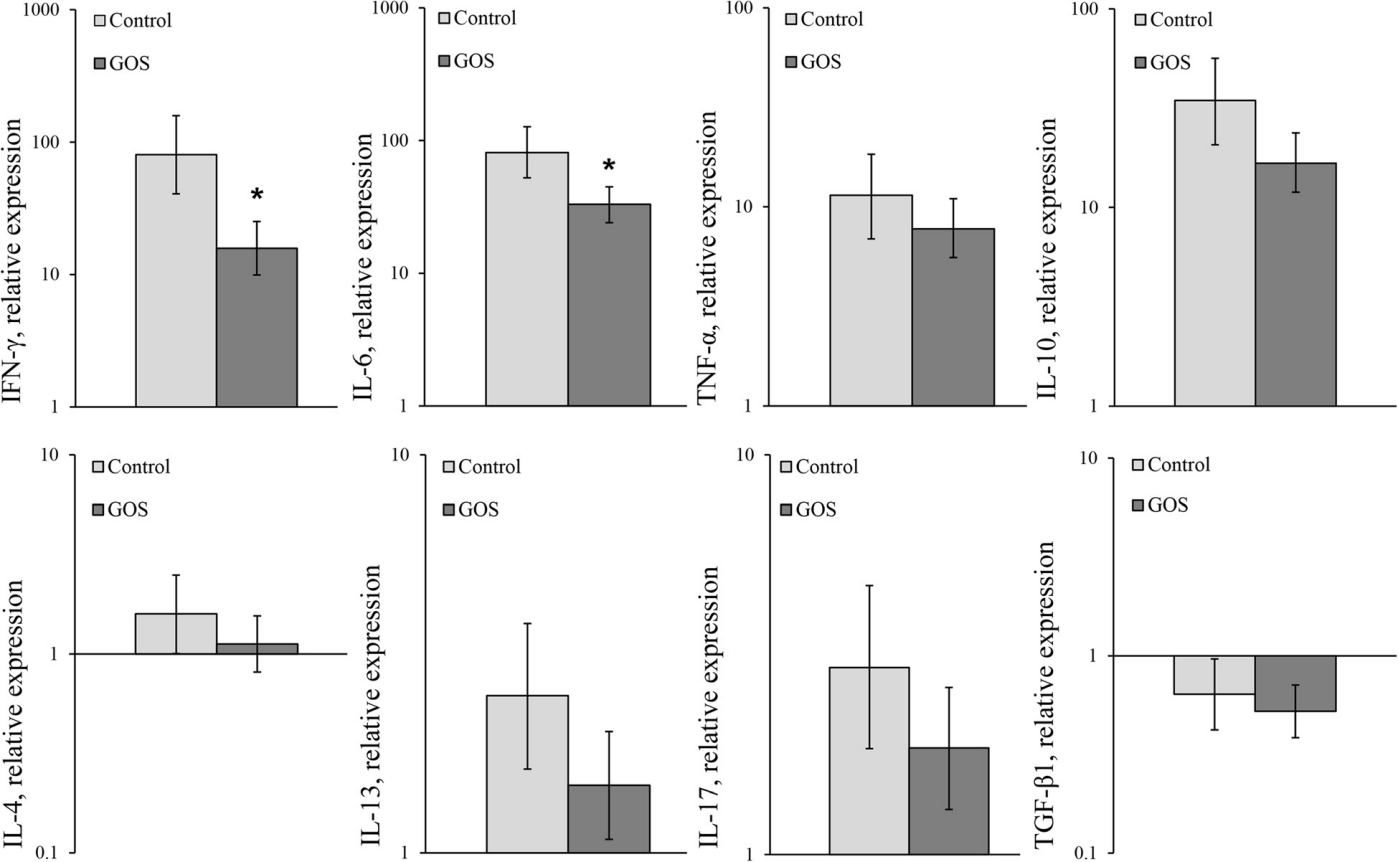
**Cytokine mRNA expression levels.** Mean relative cytokine mRNA expression levels including 95% Bayesian credible intervals in LPS-challenged PBMCs, relative to blank controls. Significant differences between PBMCs derived from foals treated with GOS and glucose/lactose (control) are indicated with an asterisk (*).

Relative cytokine expression levels after LPS stimulation in PBMCs are presented in Figure 3. In both groups, *ex vivo* LPS stimulation resulted in a significant up-regulation of mRNA expression for all investigated cytokines, except IL-4 and TGF-β1. IL-4 expression was not significantly influenced by the LPS challenge in neither of the groups, and the expression of TGF-β1 was significantly down-regulated in both groups. For IFN-γ and IL-6, the relative mRNA expression levels after LPS challenge were significantly lower in PBMCs derived from GOS-treated foals compared with the control group (80% and 40% reduction for IFN-γ and IL-6, respectively). No other significant differences were detected between the two groups.

## Discussion

In this pilot study, we found that GOS was well accepted and tolerated by newborn foals, causing no observable negative side effects. Furthermore, we did not find any significant differences between GOS-treated and control foals with regard to the investigated blood parameters. The serum globulin contents varied highly during the first weeks of life, most likely due to differences in the uptake of maternal globulins by individual foals. The measured globulin concentrations of the colostrum samples indicate that this variation was not caused by insufficient quality of the mares’ colostrum. Despite the variation in maternally acquired immunoglobulin levels, all foals eventually reached comparable and sufficient levels of immunoglobulins during the experimental period, through endogenous production (in the period >42 days of age). This active immunity of the foal itself becomes pivotal to prevent pathogens from causing disease, when maternal immunity fades [28].

At the moment of PBMC isolation, no significant differences were observed among the blood parameters of the two groups, apart from the serum concentration of IgG(a), which was significantly higher in the group of GOS-treated foals compared to the control group (see Table 2). This difference can be attributed to the particularly low serum IgG(a) concentration of the one foal with failure of passive transfer. One may be concerned about a possible influence of the serum IgG(a) concentration on the mRNA expression of cytokines in the foals’ PBMCs following LPS stimulation. If this were the case, one would expect particularly high mRNA expression rates for IFN-γ and IL-6 in the PBMCs of foals with relatively low serum IgG(a) concentrations. However, such a pattern was not present in our data; in contrast, the data showed relatively low mRNA expression levels of IFN-γ and IL-6 for the PBMCs of foals with relatively low IgG(a) concentrations. Therefore, we conclude that the differences in the PBMC experiments were probably not caused by differences in serum IgG(a) concentrations at day 28.

Our results show that the LPS-induced mRNA expression levels of IFN-γ and IL-6, which are both related to type 1 T helper cell (Th1) responses, were significantly lower in PBMCs derived from GOS-treated foals compared with the control group. However, the LPS-induced production of TNF-α in PBMCs did not differ between the two groups; similarly, there was no significant difference in mRNA expression of TNF-α. Recently, anti-inflammatory effects of oligosaccharide supplements containing GOS have been described in preterm human infants as well, though these effects were found to be transient [12]. Therefore, the PBMC data from our pilot study in foals call for future studies with PBMC experiments at multiple time points during the treatment period.

Several studies have reported measurable immunomodulatory effects of oligosaccharides through direct interaction with Toll-like receptors (TLRs) [29–31]. Moreover, transfer of oligosaccharides across the epithelial barrier was proven by Eiwegger et al.[32], suggesting that direct contact between orally supplied oligosaccharides and the cells of the immune system is possible. Taking these findings into account, we hypothesize that oral supplementation of GOS in the present study led to long-term *in vivo* interaction of PBMCs with TLRs, eventually resulting in a measurable endotoxin tolerance and less responsive PBMCs. This hypothesis is in line with our data from a previous study, in which we demonstrated direct (short-term) immunomodulatory effects of GOS on equine PBMCs *ex vivo*; co-incubation of equine PBMCs with GOS enhanced the LPS-induced cytokine responses [16]. Moreover, recently TLR-4 activation by GOS was demonstrated in several cell types [33,34]. To investigate this further, future *in vitro* experiments with repeated or long-term exposure to oligosaccharides are desirable. However, the limited viability of primary equine cells and the lack of a functional equine immortal cell line make it difficult to perform reliable experiments over a longer time frame.

Young foals have previously been shown to display lower expression levels of Th1 related cytokines, including IFN-γ and IL-6, compared with older individuals [35–37]. Immature lymphocyte responses in neonates, with a bias towards Th2 responses, are thought to play a role in the increased susceptibility to infections in early life. Therefore, immunomodulation in young individuals is generally focused on the enhancement of defensive cytokine responses instead of reducing them [38]. On the other hand, a modulated immune response to bacteria that colonize epithelial surfaces during the first period of life, including the intestines, is a prerequisite to establish bacterial colonization and concurrently maintain homeostasis [39]. Exaggerated immune responses to bacteria in the phase of colonization could influence the composition of the microbiota and even may cause tissue damage and decreased epithelial barrier function, possibly leading to bacterial infections and sepsis. If indeed GOS treatment in foals results in a lower responsiveness of gut-associated lymphocytes and dendritic cells to LPS (or other challenges), this could be an interesting finding with regard to the eventual introduction of oligosaccharide supplements in practice. Depending on the clinical context, next to disease prevention through enhancing defensive immune responses, the suppression of exaggerated cytokine responses to bacterial compounds could prevent pathology in early life, particularly in the gastrointestinal tract. On the other hand, very pronounced suppression of immune responses by GOS is not desirable as it could increase the susceptibility to infections. This question should be addressed in forthcoming *in vivo* experiments that include a pathogen challenge of treated foals. More research is required to further investigate the long-term effects of GOS, other oligosaccharides, and oligosaccharide mixtures, and to eventually optimize the use of oligosaccharide supplements in different clinical settings.

In addition, beneficial effects of oligosaccharide supplements on allergic immune responses have been reported in various mammalian species [40–45]. Recently, a Cochrane review was published on this subject, in which these significant beneficial effects are stated as well, together with the limitations of these studies and the need for more conclusive evidence [46]. The reported beneficial effects of oligosaccharide supplements in other species suggest another possible indication for the use of oligosaccharides in horses, with the aim to reduce the long-term incidence and severity of relevant immune-mediated inflammatory diseases, such as recurrent airway obstruction, inflammatory airway disease, and insect hypersensitivity (summer eczema). Further investigations into this topic are warranted.

## Conclusions

In conclusion, the results from this pilot study indicate that the chosen dose regimen of GOS was well accepted by the foals, and that no undesirable side effects were encountered. At the same time, no differences in the investigated hematological parameters, including serum immunoglobulins, could be detected between GOS-treated foals and the control group. GOS treatment appeared to reduce the pro-inflammatory responses in PBMCs after an *ex vivo* LPS challenge. With this knowledge, additional experiments with the GOS preparation can be performed in larger groups of foals, including the application of higher dosages and longer periods of treatment. Moreover, a longer follow up of GOS-treated foals may reveal long-term beneficial effects on the incidence and severity of immune-mediated inflammatory diseases, in line with published studies in other mammalian species.

## Additional file

Additional file 1:
**Investigated blood and colostrum parameters in individual foals and corresponding mares.** Included parameters are white blood cell counts (leukocytes, lymphocytes, mature granulocytes) and protein electrophoresis (total protein, albumin, α-, β-, γ-globulins) for blood samples, and immunoglobulin subtypes (IgA, IgM, IgG(a), IgG(b), IgG(T)) for blood and colostrum samples.

## Abbreviations

GAPDH: Glyceraldehyde 3-phosphate dehydrogenase
GOS: Galacto-oligosaccharides
IFN: Interferon
Ig: Immunoglobulin
IL: Interleukin
LPS: Lipopolysaccharide
PBMC: Peripheral blood mononuclear cell
TGF: Transforming growth factor
Th: T helper cell
TLR: Toll- like receptor
TNF: Tumor necrosis factor
